# Converting the existing disease surveillance from a paper-based to an electronic-based system using district health information system (DHIS-2) for real-time information: the Lebanese experience

**DOI:** 10.1186/s12913-022-07773-1

**Published:** 2022-03-25

**Authors:** Dalal Youssef, Ayat Yaghi, Abbas Jouny, Linda Abou-Abbas, Houssam Chammaa, Nada Ghosn

**Affiliations:** 1 Epidemiological Surveillance Program, Ministry of Public Health, Beirut, Lebanon; 2 Clinical Trial Program, Ministry of Public Health, Beirut, Lebanon; 3 World Health Organization, Lebanon country office, Beirut, Lebanon

**Keywords:** Disease surveillance, District health information system, DHIS-2, Paper-based, Electronic system, Lebanon

## Abstract

**Introduction:**

The Ministry of Public Health in Lebanon is in the process of converting the surveillance reporting from a cumbersome paper-based system to a web-based electronic platform (DHIS-2) to have real-time information for early detection of alerts and outbreaks and for initiating a prompt response.

**Objectives:**

This paper aimed to document the Lebanese experience in implementing DHIS-2 for the disease surveillance system. It also targets to assess the improvement of reporting rates and timeliness of the reported data and to disclose the encountered challenges and opportunities.

**Methodology:**

This is a retrospective description of processes involved in the implementation of the DHIS-2 tool in Lebanon. Initially, it was piloted for the school-based surveillance in 2014; then its use was extended in May 2017 to cover other specific surveillance systems. This included all surveillance programs collecting aggregate data from hospitals, medical centers, dispensaries, or laboratories at the first stage. As part of the national roll-out process, the online application was developed. The customized aggregated-based datasets, organization units, user accounts, specific and generic dashboards were generated. More than 80 training sessions were conducted throughout the country targeting 1290 end-users including health officers at the national and provincial levels, focal persons who were working in all public and private hospitals, laboratories, and medical centers as well. Completeness and timeliness of reported data were compared before and after the implementation of DHIS-2. The unveiled challenges and the main lessons learned during the roll-out process were discussed.

**Results:**

For laboratory-based surveillance, completeness of reporting increased from 70.8% in May to 89.6% in October. Timeliness has improved from 25 to 74%. For medical centers, an improvement of 8.1% for completeness and 9.4% in timeliness was recorded before and after training sessions. For zero reporting, completeness remains the same (88%) and timeliness has improved from 74 to 87%. The main challenges faced during the implementation of DHIS-2 were mainly infrastructural and system-related in addition to poor internet connectivity, limited workforce, and frequent changes to DHIS-2 versions.

**Conclusion:**

Implementation of DHIS-2 improved timeliness and completeness for aggregated data reporting. Continued on-site support, monitoring, and system enhancement are needed to improve the performance of DHIS-2.

**Supplementary Information:**

The online version contains supplementary material available at 10.1186/s12913-022-07773-1.

## Introduction

The public health surveillance system represents the keystone of public health practices. It was defined as the “continuous, systematic collection, analysis and interpretation of health-related data needed for the planning, implementation, and evaluation of public health practice” [1]. Given the importance of timely and accurate information for early detection of alerts and outbreaks as well as for a prompt and adequate response, a real-time consistent and coherent reporting process is crucial for an effective disease surveillance system [2]. Of note, reliable data allows public health officials to describe and accurately assess the state of health problems; hence it helps policymakers and investors to effectively allocate resources [3–6].

Given the instrumental role of data collection in any surveillance system [4] in addition to the importance of the used modality for data collection for obtaining reliable data, substantial efforts were exerted during the past few decades for improving data collection such as moving from paper-based surveillance records towards computerized electronic methods [7, 8].

In developed countries, electronic methods are used frequently for data collection, due to their advantages over the paper-based methods [9], by mitigating issues associated with paper-based methods such as avoiding processing duplicate, easing data, better completeness and timeliness, higher quality of data and greater users’ acceptance [10–12]. However, several challenges related to the collection, aggregation, compilation, analysis, and reporting of health data were encountered in developing countries [13, 14]. Moreover, limited financial and human resources, lack of sufficient training as well as the use of the traditional paper-based methods in data collection, affected the reliability and timeliness of the data [15–17] which in turn delayed the required outbreak response. Several studies highlighted various problems associated with the traditional paper-based methods use. These snags included the frequent errors occurrence, the low completeness rate, the duplication issues, and the storage costs as well [18, 19]. Therefore, many developing countries were focusing currently on moving their reporting systems from paper-based methods to electronic methods [20–22].

Among the web-based systems used in developing countries, the District Health information system (DHIS-2) was one of the most popular tools. This free and open-source platform open was developed by the Health Information Systems Program (HISP) at Oslo University. Used by multiple organizations [23], it served as a “tool for collection, validation, analysis, and presentation of aggregate statistical data, tailored but not limited to integrated health information management activities [24, 25]. Thus, it facilitates analysis of collected data across all the public health facilities that enhances forecasting of required services for future planning purposes and evaluating the performance of healthcare workers [18]. DHIS2 has several features and functionalities. It provides a comprehensive health information system for the reporting and analysis needs of users at analysis at different levels of organizational hierarchy. It can easily be customized to the different requisites of the information system through the user interface without need of programming to start using this tool in a new setting. In addition, it provides data entry tools able to replicate the paper forms as well as data validation tools allowing the improvement of data. Besides, it enables flexible and dynamic data analysis in the analytics modules and one-click reports with charts and tables for selected indicators. The availability of a user-specific dashboard allows quick access to the relevant monitoring and evaluation tools. It has also the functionality to manage user’s account and role and to export-import of data and metadata. Lastly, it represents an active information-driven user community as it allows users can share and discuss their data in charts and reports. Since its release in 2006, DHIS-2 was used by non-governmental organizations (NGOs) and national governments in more than 60 countries where it was deployed for health-related projects, including monitoring of patient health, improving disease surveillance, pinpointing outbreaks, and speeding up health data access [26].

In Lebanon, the surveillance system has been reactivated after the civil war in 1995, by the ministry of public health to monitor infectious diseases across the country [27]. Since then, most cases are reported via fax from different data sources to the epidemiological surveillance unit (ESU). In respect of hierarchy, data flow should pass by several steps before reaching the national level which resulted in a delay in receiving information and thus in outbreak detection and response. Automated reporting from data sources directly has been suggested as means to improve the quality of data and timeliness of disease notification and response [28, 29]. In 2016, the assessments of the surveillance system conducted in Lebanon (Early warning system EWARS mission and joint external evaluation JEEE) highlighted the importance of moving from a paper-based system and recommended the deployment of a web-based reporting system able to provide real-time information [30]. Such migration process required a web-based reporting system using a standard tool compatible with various devices (portable computers, tablets, smartphones, and android), linked with dashboards and alert systems, and supported by international academia. In this context, the DHIS-2 tool was selected to fulfill needed functions. Lebanon was one of the first Eastern Mediterranean countries to adopt a bottom-up strategy to implement DHIS2. The latter was done by first introducing the use of DHIS2 for a specific program and then incrementally expanding to also include other programs (case-based surveillance for communicable diseases). In 2017, the Ministry of Health in Lebanon started the process of migrating surveillance reporting from a cumbersome paper-based system to a web-based electronic platform (DHIS-2). This project aimed to provide real-time information to timely detect alerts and outbreaks. Given the importance of the documentation in ensuring the fulfillment of the project requirements and the establishing of traceability, it is of great interest to document the Lebanese experience in implementing the DHIS-2 for diseases surveillance during this transitory phase and to highlight the opportunities and the challenges encountered during this experience. This article will serve as a reference for the implementation of future functionalities and for system use’ extension across the country.

Therefore, this paper aimed to document the Lebanese experience in implementing DHIS-2 for the disease surveillance system. It also targets to assess the reported data completeness and timeliness improvement and to disclose the encountered challenges and opportunities.

## Methods

This is a retrospective description of processes involved in the implementation of the DHIS-2 tool in Lebanon.

### Pilot phase

Before initiating the national system roll-out, the DHIS-2 tool was initially piloted in Lebanon in 2014 for school-based absenteeism monitoring surveillance using dedicated national servers. Testing of the usability of this tool was performed to evaluate the potential users’ acceptance of the system allowing incorporating of adjustments promptly. Targeted end users were epidemiological surveillance staff. They would be the backbone for DHIS2 implementation. They were requested to provide their feedback in regards to their data entry experience including their insights about the designs of data entry forms, the flexibility, and the data entry easiness as well as the online data entry feasibility.

### DHIS-2 customization and setup

Before the full-scale implementation of DHIS-2, there was a need to customize the tool to suit the surveillance needs. First, we define data elements that are considered the most important building block of a DHIS-2 database. Data elements represented the “what dimension” to be collected or analyzed. A dictionary of data elements for each reporting form was created. Since DHIS2 with its open metadata model and a flexible user interface allows the user to design the contents of a specific information system without the need for programming, aggregated-based datasets were customized according to the format of each paper reporting form. The customization referred to the creation of an electronic form that retained the same format and variables as the paper form and had flexibility in design. Of note, such customization took into consideration additional factors such as the hardware used to perform data entry as some facilities may use also mobile devices to perform data.

The customized data sets were as follow: the hospital zero reporting form, medical centers reporting form, and laboratory reporting forms.

#### The hospital zero-reporting form

the zero-reporting system was first established for acute flaccid paralysis (AFP) surveillance in order to enhance the awareness of health institutions on the importance of case detection and reporting. Based on the MOPH decision number 1162/2 dated 5 December 2001, all hospitals in both public and private sectors were required to adopt the zero-reporting system which included all immediately notifiable communicable diseases. The zero-reporting system is both passive (for MOPH) and active (for the hospital). At the hospital level, the designated focal person searches for cases in the wards and fills the zero-reporting form, then sends it on weekly basis to the MOPH surveillance team at the district level. The zero-reporting form includes the following data: hospital name, week identification (starting on Monday), number of cases for each target event, and reporter identification and contact details (Annex 1).

#### The medical center reporting form

which is a weekly aggregated data-based, included variables divided into the following categories: general identification variables, reportable health events, referral for patients, or deaths. A detailed description of the variables included in the medical center and dispensary-based surveillance system was provided in Annex 2.

#### The laboratory-based surveillance form

the laboratory-based surveillance system was launched nationally in all Lebanese provinces in 2013 based on the MOPH decision no. 315/2 that requests all laboratories in Lebanon in public and private sectors, in-hospitals, and outside hospitals, to be part of the new surveillance system and to report on a weekly basis through an aggregated-based laboratory form. This form is divided into the following categories: Laboratory general information, bacteriological culture, other stool analysis, Serology, and influenza (Annex 3). Based on routine clinical laboratory tests, this system provides real-time early warning information to decision-makers about infectious diseases. It is a tool to monitor the trends of communicable diseases, detect outbreaks, and implement effective control measures. Of note, all these forms were reported on a weekly basis from different data sources (hospitals, medical centers, and laboratories).

After generating the mentioned data sets, organization units hierarchy was established and 5 levels were defined as follows: national (level 1), provincial (level 2), district (level 3), locality (level 4), and facility (level 5). The target data sources were: medical centers, mobile clinics, hospitals, and laboratories. Each facility is an orgunit identified within the organization hierarchy. For each organization unit, users’ accounts were created specifying the username, password, and accessibility. Access profile is defined according to the user role. It allows data sources to perform data entry, add, update and analyze their data using their computers or mobile. DHIS2 dashboards were introduced for data visualization on an everyday basis and two types of dashboards were generated: specific and generic dashboards. Dashboards are intended to provide quick access to different analytical objects (maps, charts, reports, tables, etc.…) to an individual user and can be shared with user groups and could serve as a monitor tool. Each facility had access to its dashboard which is a generic one. MOPH users had access to the generic and specific dashboards. To ensure data entry accuracy and to improve data quality, validation rules were created. Of note, data quality referred to the completeness, timeliness, and correctness of data. Poor data quality can take many shapes; not just incorrect figures, but a lack of completeness, or the data being too old (for meaningful use). The validation during data entry to make sure data is captured in the right format and within a reasonable range, user-defined validation rules based on mathematical relationships between the data being captured (e.g. subtotals vs totals), outlier analysis functions, as well as reports on data completeness. In terms of data input validation which is the most basic type of data quality check in DHIS2, it aimed to make sure that the data being captured is in the correct format. When a wrong format was entered, the DHIS2 gives the users a message that the value entered is not in the correct format and will not save this value until it has been changed to an accepted value. In addition, minimum and maximum ranges for the value were defined based on the previously collected data for the same data element. Entered data were checked if the value being entered is within the reasonable defined range. The users were alerted as soon as they entered a value outside the accepted range. In addition, validation rules were established, based on an expression that defines a relationship between a number of data elements. When running validation rules, and checks have been completed, a report presenting validation violations explains which data values need to be corrected. Outliers can occur by chance, but they often indicate a measurement error or a heavy-tailed distribution (leading to very high numbers).

### National roll-out and training sessions

After the official decision to implement DHIS-2 at the data sources level, 80 training sessions targeting 1290 users were conducted throughout the country including two rounds of training for laboratories, two rounds for medical centers, and one round for hospitals. The training material includes a combination of theoretical lectures and hands-on practice. Trained people included district and provincial health officers, 150 designated focal persons working in all public and private hospitals, 140 focal persons from laboratories, and 800 focal persons working in medical centers and dispensaries. In addition, a user guide was developed in three languages (English, French, and Arabic) and distributed to data sources during training. A standardized training curriculum and tools are also needed. Furthermore, soft copies of training manuals were shared with staff during training sessions. Given the different data sources where DHIS-2 was used, the DHIS-2 training curriculum was tailored to the needs of health professionals working at different facilities. Subsequently, a circular issued by the ministry of health requested data sources to start reporting their aggregated data to the epidemiological surveillance program using the DHIS-2 platform.

### Data flow

**Fig. 1:**
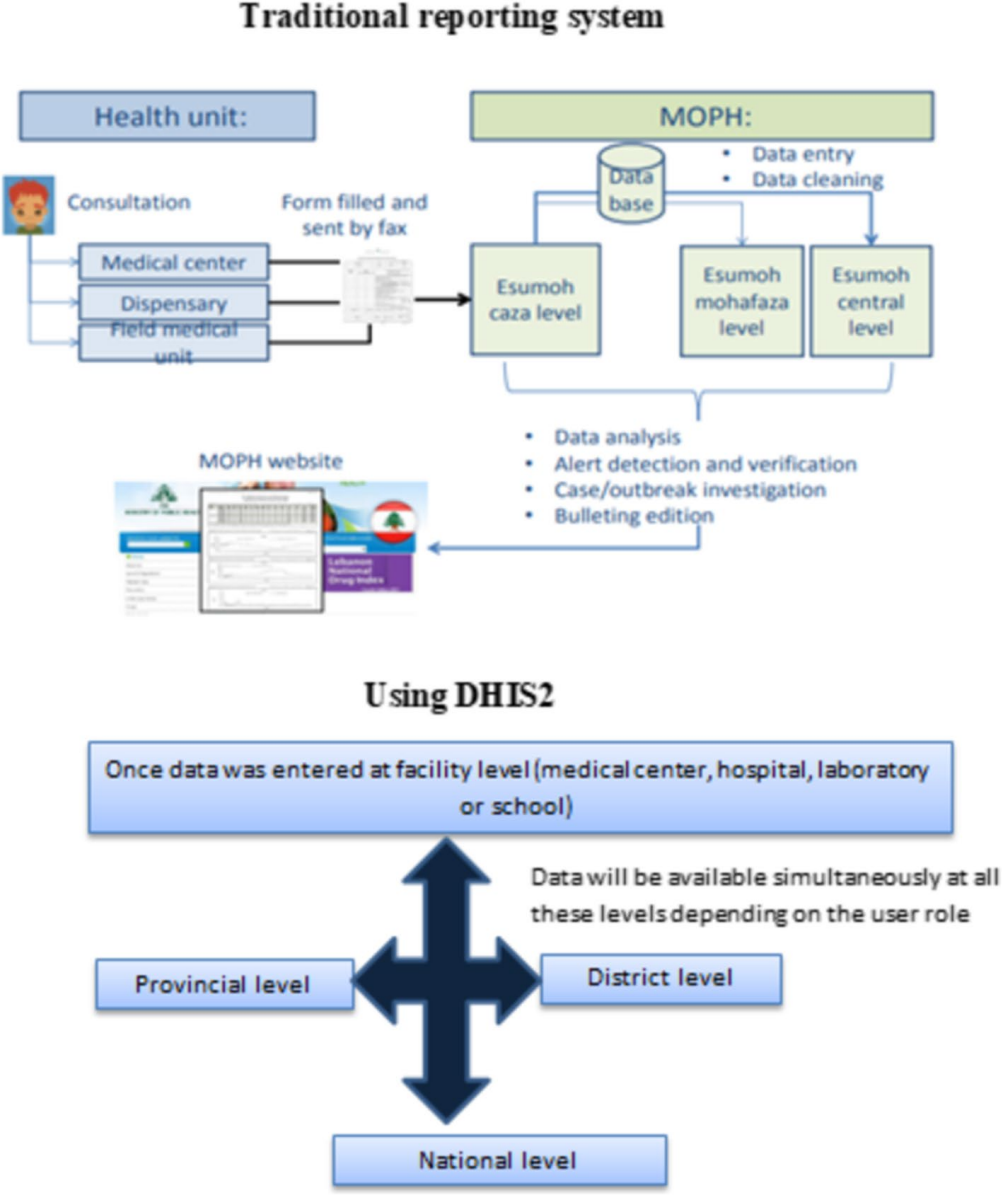
Data flow

Data were entered at data sources using DHIS2, then it became available and accessible timely by users depending on their defined roles. Of note, the information was simultaneously available at different hierarchical levels of the system (Fig. 1). Alerts generated by fixed thresholds, relative increase or historical data were detected at peripheral level (district and provincial levels) where ESU staff conduct first verification by contacting the health facility. In case of verified alert, an investigation is launched. This process is done by the ESU districit team supported by the ESU provincial and central team.

### Indicators

Indicators such as completeness and timeliness were computed. Weekly reporting completeness was defined as the proportion of health units that have reported for a specific week among the total number of health units. It was calculated using the following formula:

$$Weekly\ reporting\ completeness=\frac{number\ of\ received\ for ms\ for\ one\ specific\ week\ast 100}{number\ of\ expected\ reporting\ for ms\ for\ that\ specific\ week}$$

As for weekly reporting timeliness, it was defined as the proportion of health facilities reporting on time for a specific among the total number of health units expected to report for that specific week and it was calculated as follow:

$$Weekly\ reporting\ timeliness=\frac{Number\ of\ forms\ received\ on\ time\ for\ on e\ specific\ week\ast 100}{number\ of\ expected\ forms\ for\ that\ specific\ week}$$

To assess improvement in data reporting, a comparison of the completeness and timeliness of data before and after the implementation of DHIS2 was performed. Of note, pre-implementation data were obtained from the traditional surveillance system managed by the ESU that used the paper-based reporting method to collect data from data sources (hospitals, laboratories, and medical centers).

## Results

Our results showed an improvement in both completeness and timeliness of data reporting following the implementation of DHIS-2 in our data sources: laboratories, hospitals, and medical centers.

### For laboratory surveillance

In 2017, a total of 6626 forms were received from all laboratories working inside the hospitals across Lebanon. On average, 120 forms were received on weekly basis. The annual average completeness rate was 78.8%. Starting week 18 of 2017, an improvement in the completeness was registered which coincide with the roll-out of DHIS-2 for laboratory-based surveillance.

**Fig. 2:**
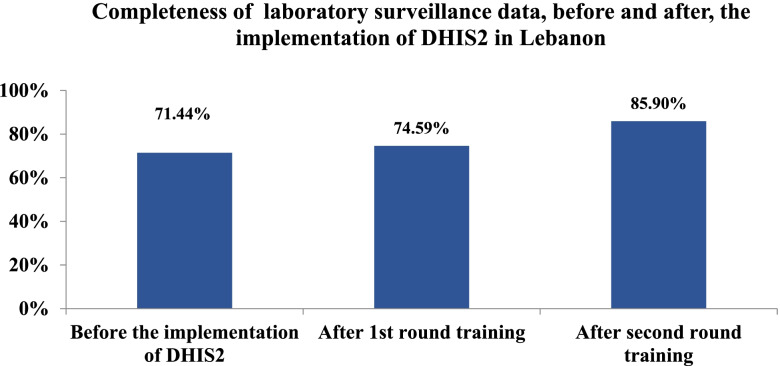
Completeness of laboratory surveillance data before and after the implementation of DHIS2 in Lebanon

For the period extended from week1 to week 18 in the year 2017, data was received via fax and was entered by the epidemiological unit staff at the peripheral level using DHIS-2. At week 18, the average completeness of laboratory surveillance was 71.4%, it increased to 74.59% after the first round of training for end-users and reached 85.9% after the second round (Fig. 2).

Table 1 displayed the completeness of laboratory-based surveillance data, before and after, the implementation of DHIS-2 across all provinces. Four provinces registered an average completeness weekly rate equal to or above 90% after the second round of training.

**Table 1:** Completeness of laboratory surveillance data, before and after, the implementation of DHIS-2 in Lebanon

	Akkar	Baalbeck-Hermel	Beirut	Bekaa	Mount-Lebanon	Nabatyeh	North	South	Lebanon
Phases									
Before the implementation of DHIS-2	1.18%	91.70%	32.74%	98.18%	67.88%	88.24%	70.35%	88.85%	71.44%
After 1st round training	45.45%	89.41%	40.93%	100.00%	73.64%	90.00%	64.02%	87.56%	74.59%
After second round training	65.83%	91.70%	63.83%	96.36%	81.00%	97.92%	89.75%	94.95%	85.90%

The improvement of completeness of laboratory data was detected in all provinces. The highest completeness rate was recorded in Nabatyeh (97.92%) followed by Bekaa province (96.36%). A noteworthy improvement was revealed in Akkar (64%) and Beirut (31.1%) provinces. For Akkar province, a very low completeness rate (1.18%) was recorded before the DHIS2 implementation. However, this completeness increased to 45.45% after the first round of training and reached 65.83% after the second round. Similarly, the completeness reached 63.83% for Beirut province after the second round (Table 1).

**Fig. 3:**
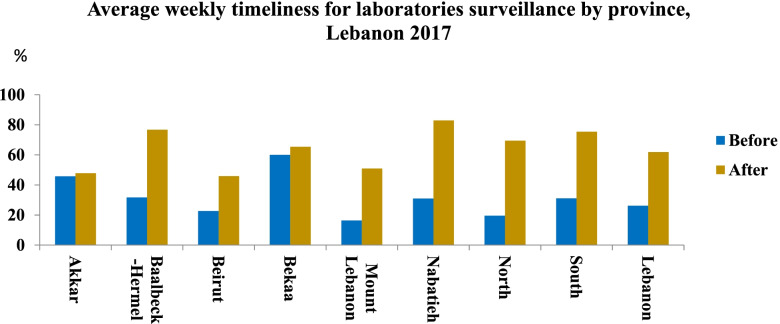
Average weekly timeliness for laboratories surveillance system by province, Lebanon 2017

In terms of timeliness, an increase from 26.18% before to 61.87% after DHIS-2 use was registered at the national level. This improvement was also shown in all provinces. The highest timeliness rate was recorded in Nabatyeh (82.92%), followed by Baalbeck-Hermel (76.76%) and South (75.43%) provinces (Fig. 3).

**Fig. 4:**
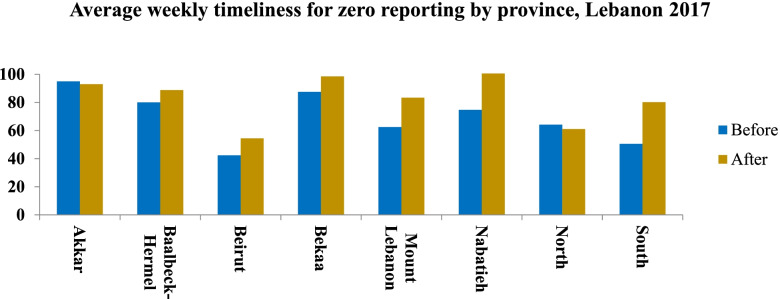
Average weekly timeliness for zero reporting by province, Lebanon 2017

Table 2 displayed the average weekly completeness (AWC) rate before and after use of DHIS-2, in Lebanon for the year 2017. For the zero reporting system, the completeness decreased slightly from 95.54% before to 88.79% after the use of DHIS-2 but still ranked at a good level. However, a completeness improvement was recorded in Nabatyeh (99.41%) and South (98.14%) (Table 2). Timeliness was also improved markedly. It reached 79.79% after the use of DHIS-2 at the national level with an important improvement in timeliness of zero reporting registered in all provinces (Fig. 4).

**Table 2:** Average weekly completeness (AWC) rate before and after use of DHIS-2, Lebanon 2017

		AWC rate Before	AWC rate After
**L**aboratory surveillance	**Lebanon**	**71.46**	**82.66**
	Akkar	20.00	60.59
	Baalbeck-Hermel	92.15	90.96
	Beirut	31.97	57.75
	Bekaa	98.28	97.43
	Mount Lebanon	68.00	78.94
	Nabatieh	88.33	95.59
	North	70.37	81.98
	South	88.88	92.72
Medical centers	**Lebanon**	**47.08**	**58.22**
	Akkar	21.23	43.69
	Baalbeck-Hermel	21.82	54.91
	Beirut	2.59	40.71
	Bekaa	83.66	77.65
	Mount Lebanon	47.15	52.29
	Nabatieh	68.99	79.30
	North	34.45	49.94
	South	58.20	68.89
Zero reporting	**Lebanon**	**95.54**	**88.79**
	Akkar	97.71	92.94
	Baalbeck-Hermel	91.45	91.21
	Beirut	86.99	76.49
	Bekaa	99.82	99.27
	Mount Lebanon	96.60	93.24
	Nabatieh	97.71	99.41
	North	94.87	64.22
	South	96.84	98.14

**Fig. 5:**
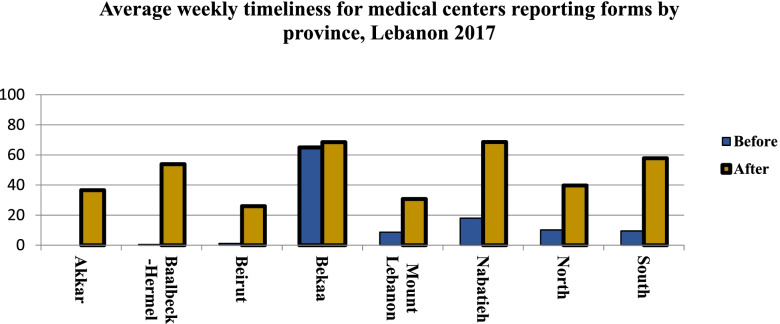
Average weekly timeliness for medical centers reporting forms by province, Lebanon 2017

For medical centers: An improvement in the completeness and timeliness was recorded at national and provincial levels (Fig. 5). At the national level, the completeness rate was 47.08% before DHIS-2 use; it reached 58.22% after DHIS-2. Similarly, for the timeliness, an increase from 9.52 to 45.15% was recorded (Table 2).

### Implementation challenges

Several operational challenges, mainly related to infrastructure and human resources, were encountered during the implementation of DHIS-2 in Lebanon. First, the proper communication infrastructure, revealed by limited access to internet services and poor internet connectivity particularly in peripheral areas combined with a scarcity of available electronic devices (computers, tablets….) which makes real-time data entry difficult to a certain extent.

Other common key challenges were related to human resources (both numbers of personnel and their ability, knowledge, and experience) such as skilled personnel availability and shortage of adequately trained personnel. Internet illiteracy and limited skills in using electronic devices among several end-users were identified as barriers for DHIS-2 implementation in Lebanon. Given that DHIS-2 success relied on human capacity, a challenge of staff turn-over and redeployments occurred commonly at data sources (medical centers and laboratories) and the newly recruited staff lacked knowledge and training. However, errors in data entry continue to occur but less frequently than paper reporting as validation rules were created.

Another key challenge in the implementation of DHIS-2 was the frequent DHIS2 versions changes. Lastly, it is worth mentioning that the DHIS2 was based on the premise of open data, therefore, this feature of the software has created security concerns.

### Lessons learned

Several lessons learned during the implementation phase in Lebanon that should be taken into consideration are listed below:

- Having the highest level endorsement for DHIS2 implementation at the MOPH
- Planning and financing of DHIS2 implementation are crucial for the success and sustainability of such a project.
- Training of many focal persons in each data source
- Allowing users to change regularly their credentials (passwords) for security reasons.
- Data ownership was critical for the success of such implementation
- Ensuring privacy and security of the platform
- Customizing and formatting the dashboard to make it more interactive.
- Strict monitoring and defining the staffs’ role were also recommended
- Establishing a monitoring and evaluation framework
- Regular monthly review meetings should be made to track improvements in performance
- Performing refresher training after every update to the software or data reporting forms

## Discussion

The present paper aimed to document the Lebanese experience in implementing DHIS-2 for disease surveillance in Lebanon. It emphasized strengths in both technical and operational features of DHIS2 and shed light on challenges and concerns encountered during the implementation as well.. Our experience showed that the use of DHIS-2 in surveillance improved timeliness, completeness, and data quality. DHIS-2 reduced bureaucratic delays associated with paper-based use in surveillance and data is available in all hierarchical levels upon completion of data entry by the data sources. Several challenges were encountered during the implementation of DHIS-2 in Lebanon, mainly related to infrastructure and human resources.

This improvement in data reporting showed in our implementation experience could be due to the several training performed by ESU for focal persons working in targeted data sources. The regular follow-up performed by ESU peripheral and central teams has played a crucial role in this improvement as well. A huge effort was exerted by the ESU to monitor the process and to support data sources in the initial steps of using DHIS-2. They were also ready and available to assist in case needed. Health officers at the district level conducted also on-hand training for the focal persons, working in data sources, who faced difficulty in performing data entry. In addition, the mandatory quality checks created at different levels have played a significant role in improving data quality where incorrect values were not allowed to be entered. This was due to the rules generated at the data entry level which were based on input validation, identifying reasonable ranges, and outliers. Of note, the generation of the dashboards makes DHIS2 compatible to use as a monitoring tool. Our findings were in line with the improvement reported by other countries that implemented a web-based data quality intervention. For example, South Africa has witnessed a significant improvement in data completeness from 26% before to 64% as well as overall accuracy of data that increased from 37% at the first data audit to 65% (22).

In terms of reducing data entry errors, our results were in line with the findings of a study of the impact of a web-based reporting system on the collection of medication error occurrence data in the United States, where Rudman et al. found that the missing or unspecified data from the cause-of-error variable decreased from 18.6 to 2.1% [10]. It is worth mentioning that verification of data entry errors was swifter since it was directly linked with data sources. In addition, ownership of data by DHIS-2 users’ and their accessibility to their data could increase their responsibility feelings in terms of data quality and encourage them to produce good quality data in terms of correctness, completeness, and timeliness. In this way, the attributes of the DHIS2 system enhance a decision-making culture in relation to the use of data. Therefore, DHIS2 represented a dynamic system that could improve the overall accountability of data reporting from data sources.

In terms of economic benefits, DHIS2 could help in the reduction in costs associated with paper use for data reporting for the mentioned forms. Prior to the implementation of DHIS2, data sources including hospitals, laboratories, and medical centers (> 1000 facilities) were used to report their weekly data through fax to the ESU peripheral level (district level), then the peripheral team, in turn, send it to the provincial level through fax. The latter will repeat the process to share the data with the ESU central level. The data flow consumes time and paper. Sometimes, forms were transported to the ESU team in case there is a glitch in the fax.

As for completeness and timeliness, our results showed that these indicators were notably improved for medical centers and laboratories’ reporting systems. However, this was not the case for the zero reporting system where completeness seems to slightly decrease after the use of DHIS-2. This could be due to the restrained users’ familiarity with this platform which is usual during this transition phase. In addition, zero reporting forms were previously actively collected and followed by the MOPH staff responsible for a field visit to hospitals. Our results were in line with many studies conducted in Uganda, Kenya, and South Africa which emphasized an improvement of reporting rates and timeliness of data. All these countries have shown an improvement in reporting rates after the adoption of DHIS-2 [22, 31, 32].

Furthermore, the use of DHIS2 contributes to the development of the data management culture. This will be resulting from increased access to information that supports and protects transparency. In addition, the DHIS2 tool has the ability to properly analyze and visualize data and to generate reports as well, therefore providing feedback to data sources. One additional benefit of the use of DHIS2 for health services is the flexibility of the software allowing users to make modifications based on their needs. Health facilities could also suggest new modules to be added. Thus, DHIS2 can be honed to the local needs of each country and each surveillance system.

Despite the above-mentioned benefits of the use of the DHIS2 tool, the implementation of DHIS2 in Lebanon has encountered several challenges similar to those faced by other countries. Recognizing these issues can help policymakers make better decisions. DHIS2 like other web-based information systems has the potential to operate without a local software installation. Although the bandwidth required for users to enter data into the DHIS2 server is modest, such a platform required some amount of internet connectivity. In addition, the data entry required hardware (computers, laptops, or smartphones) and electricity which may not be available in all health facilities. Furthermore, these tools could be shared by other programs which makes them not accessible. Based on our results, limited access to internet services and poor internet connectivity particularly in peripheral areas combined with a scarcity of available electronic devices (computers, tablets….) were found. Hence, there is an urgent need to improve the information and communications technology infrastructure required to support the implementation of this application. Similar challenges were found by the experiences of other countries, in terms of proper communication infrastructure.

Furthermore, internet illiteracy and limited skills in using electronic devices among several end-users could lead to reluctance and lack of motivation to use the new electronic system by these end-users. Addressing the reluctance of end-users through education, continuous training, smooth adaptation to technology, and respecting time needed to be familiar with an electronic system are recommended [33].

Our findings in terms of human resources challenges were in line with a study conducted in Ghana where limited knowledge of the use of the DHIS-2 software hindered the implementation of such a tool [34]. Since DHIS-2 success relied on human capacity, therefore, there is a need to develop and enhance users’ skills through regular training on DHIS-2. To address the turn-over and redeployments that occurred commonly at data sources, refresher training is recommended in addition to a regular update of passwords for security reasons.

Another key challenge in the implementation of DHIS-2 was the frequent change in DHIS2 versions. Lastly, since DHIS2 was based on the premise of open data, security concerns are key issues that should be also taken into consideration. Such a feature of the software has created concerns also in some countries. In this regard, allocating supportive strategies are required.

Of note, this shared experience is based on a short duration of DHIS-2 implementation. Some listed challenges could result from limited familiarity with the new information system during the transition phase.

In terms of lessons learned during the implementation phase, having the highest level endorsement for DHIS2 implementation at the MOPH which falls under “political leadership” was crucial for the success of such implementation. The DHIS2 platform ownership and leadership by the MOPH ensure that official documents and regulations are primed. Of note, the issuing of several official documents that recognized DHIS2 has enabled a smooth rollout of the implementation process. For example, the circular issued by the MOPH stated that the epidemiological surveillance program would implement DHIS2 countrywide and specified policy guidelines for unified data collection, data flow policy, and implementation guidelines. The latter was a strong signal for data sources (medical centers, hospitals, laboratories...) to be involved and to report the required data through DHIS2. The MOPH represents the ownership leading the standardization process and coordinating with data sources and the WHO country office provides technical support competence. The coordination and the collaboration between the ESU team and the focal persons at data sources have played a crucial role in the process of implementation in terms of supporting the capacity building for the data sources focal persons and enhancing the acceptance of the new system.

To enhance the effectiveness and sustainability of the DHIS2, planning and financing of DHIS2 implementation and capacity building of the national central team and subnational teams for provincial and district levels are critical. This included a detailed identification of human, financial, infrastructure, and technical needs. Of note, a clear definition of role and responsibilities was also highly required as it will allow the ESU peripheral team to be able to manage DHIS2 at their level, support the district team, provide reports, dashboards, and support data sources to use their own data.. National and provincial teams play a crucial role in ensuring data quality and that the data are being used and that the system is managed and adapted to the needs of the users.

Furthermore, education and training are keys to rendering the system sustainable and convenient; the ESU team should regularly conduct refresher courses in DHIS2 for grass root focal persons at data sources so as to build the capacity to effectively manage data using the tool. Refresher training after every update to the software or data reporting forms, to improve end users’ skills and efficiency is required. In addition, it is recommended to Train many focal persons in each data source as a preventive measure. This aimed to limit challenges related to work absenteeism of trained personnel and staff turn-over which could impede the sustainability of the work.

Despite that the DHIS2 platform is free, the customization of forms and dashboards and the introduction of additional functionality as well required significant resources. Hence, it is important to establish a DHIS2 support team that consists of software specialists who could support any additional required customization of DHIS2 to surveillance needs. It is recommended that this team collaborate with the University of Oslo and HISP.

It is noteworthy that enhancing the ownership of data by facilities in addition to generating interactive dashboards are critical for the success of such implementation since giving access to data sources to their data and their results encourage their involvement in the system and enhance their sense of responsibility.

Lastly, monthly review meetings should be made to track improvements in performance through comparison of the previous month, present month, and yearly national targets. The latter could help to identify any hindrances to achieving targets.

## Future directions

Before expanding the use of DHIS-2 for case-based surveillance, an extensive and comprehensive evaluation of DHIS-2 implementation is recommended. Establishing a monitoring and evaluation framework to identify DHIS2-related facilitating factors at all steps ranging from input, process, and outcome is also required. Concerning users’ experience with DHIS-2 implementation, there is a lack of clear barriers and enablers encountered by DHIS-2 users. Given that acceptance of the new system by end-users and their satisfaction are pillars for the success of such implementation, there is a need to assess the acceptability of the DHIS-2 system as an innovation in Lebanon and to understand the key determinants of this acceptance. Besides, an assessment of the overall satisfaction of end-users with DHIS-2 is recommended.

## Conclusion

After 6 months of DHIS-2 use in disease surveillance of aggregate-based data, reporting rates and timeliness of reporting of aggregated data were improved. Errors and bureaucratic delays associated with paper-based systems were minimized as well. The data quality was enhanced and the data management was eased by using an accessible data repository. To fully harness the potential of DHIS-2, the identified challenges must be addressed through continued onsite support, monitoring, and system enhancement.

## Supplementary Information

**Additional file 1.**

## Data Availability

The data supporting study findings are owned by the Epidemiological Surveillance Program (ESU) in the Lebanese Ministry of Public Health. Restrictions may apply to the availability of these data. However, data are available from the authors upon reasonable request after getting the permission of the head of the epidemiological surveillance program head coordinator esumoh@moph.gov.lb. A proposal with a detailed description of study objectives and a statistical analysis plan will be needed for the assessment of requests.

## Abbreviations

DHIS-2: District Health Information system-2
ESU: Epidemiological surveillance unit
MOPH: Ministry of Public Health
EWARN: Early warning system
JEE: joint external evaluation
HISP: Health Information Systems Program
ICT: Information and Communications Technology
WHO: World Health Organization
NGOs: Non-Governmental Organizations
