# Supplementary material for: Converting the existing disease surveillance from a paper-based to an electronic-based system using district health information system (DHIS-2) for real-time information: the Lebanese experience
Source: BMC Health Serv Res. 2022 Mar 25;22:395. doi: 10.1186/s12913-022-07773-1 (PMC8957192; doi:10.1186/s12913-022-07773-1)

Annex 1:Zero-reporting form

الجمهورية اللبنانية
وزارة الصحة العامة

تلفون:

فاكس:

جانب برنامج الترصد الوبائي
الموضوع: الإبلاغ الصفري الأسبوعي Zero-reporting للأمراض الانتقالية ذات الإبلاغ الفوري

المرجع: مستشفى _
الاسبوع: من الاثنين _ _ _ _ _ _ _ _ _ _ إلى الأحد _ _ _ _ _ _ _ _ _ _

خاص لوزارة الصحة العامة

تاريخ الاستلام
رقم الاستمارة

عدد الحالات المشتبهة / المثبتة هي:

Other immediate notifiable diseases/ أمراض انتقالية اخرى ذات إبلاغ فوري: Anthrax, Diphtheria, Food poisoning, Hemorrhagic fever, Mumps, Pertussis, Plague, Rabies, Smallpox, Tetanus, Unusual/unexpected event	Novel respiratory viruses/ الفيروسات التنفسية المستجدة : Novel Influenza Viruses انفلونزا ناجمة عن نمط جديد Or Novel Coronavirus: SARS, MERS-COV / فيروس التاجي المستجد	Cholera / الكوليرا	Measles & Rubella & Congenital Rubella Syndrome/ الحصبة والحصبة الألمانية والحصبة الالمانية الخلقية	Meningitis (Bacterial, viral) التهاب السحايا الحاد Or Invasive meningococcal disease	Acute Flaccid Paralysis / الشلل الرخو الحاد أيا كان التشخيص الطبي لدون ١٥ سنة، بما في ذلك: Guillain Barre syndrome, transverse myelitis, acute neuritis … Or Acute Poliomyelitis / شلل الاطفال	
						قسم طب الأطفال<br>اسم الطبيب<br>- - - - - - - - - -
						قسم الطب الداخلي<br>اسم الطبيب<br>- - - - - - - - - -
						قسم العناية الفائقة<br>اسم الطبيب<br>- - - - - - - - - -
						قسم الطوارئ<br>اسم الطبيب<br>- - - - - - - - - -

اسم ضابط الاتصال: _ _ _ _ _ _ _ _ _ _ _ _ _ _ رقم الهاتف: _ _ _ _ _ _ _ _ _ _ _ _ التوقيع:

Annex 2: Variables included in the medical center and dispensary based surveillance system form

Categories	Variables
**General identification**	- Medical center / dispensary / field medical unit name<br>- Location: mohafaza, caza and locality<br>- Identification of the week, starting on Monday
**Reportable health events**	Target health events are classified by two age group (>5 and <5).<br>**For vaccine preventable diseases:**<br>- Number of acute flaccid paralysis cases<br>- Number of measles cases<br>- Number of rubella cases<br>- Number of pertussis or whooping cough cases<br>- Number of mumps cases<br>**For other communicable diseases:**<br>- Number of acute diarrhea cases<br>- Number of bloody/dysenteric diarrhea cases<br>- Number of cholera cases<br>- Number of acute jaundice cases<br>- Number of acute respiratory infection and flu-like illness cases<br>- Number of unexplained fever cases<br>- Number of scabies cases<br>- Number of leishmaniasis cases<br>- Number of cases related to other mandatory notifiable diseases<br>- Number of cases during outbreak<br>**Others**<br>- Number of asthma cases<br>- Number of accidents/injuries cases<br>- Total number of other consultations
**Inpatients**	In case of referral to hospital, the following varialbes are specified: name, age, gender, locality, hospital name and medical diagnosis.
**Deaths**	In case of death in the health structure, the following variables are specified: name, age, gender, locality and cause of death.

Annex 3: Laboratory form categories and variables

Categories	Variables
**Laboratory general information**	- Laboratory name<br>- Director name<br>- Laboratory register number<br>- Identification of the week, starting on Monday
**Bacteriological culture**	- Bacteriological culture in CSF, blood, stool and respiratory specimen: total done, total negative, total positive<br>- Total positive for the following: Brucella, Campylobacter, Cholera, E. Coli, Haemophilus influenza, Listeria , Neisseria meningitidis, Salmonella, Shigella, Streptococcus pneumonia, Streptococcus and others
**Other stool analysis: direct exam and rapid test**	- Direct stool exam: total done, total negative, and total positive<br>- Total positive for: Entamoeba histolytica, Giardia lamblia and others<br>- Stool EIA Rotavirus antigen detection: total done, total negative and total positive
**Serology**	- Specific serological tests for hepatitis A virus, Measles, & Rubella: total done, total negative and total positive
**Influenza**	- Influenza rapid test, in particular for A and B: total done, total negative, and total positive<br>- PCR Influenza test, in particular for Influenza A, B A(H1), A(H3), A(H5) and others
**Notes**	- Remarks<br>- Name and signature<br>- Date

Annex 4: Example of specific dashboard and generic dashboard

Specific dashboard for MOPH team

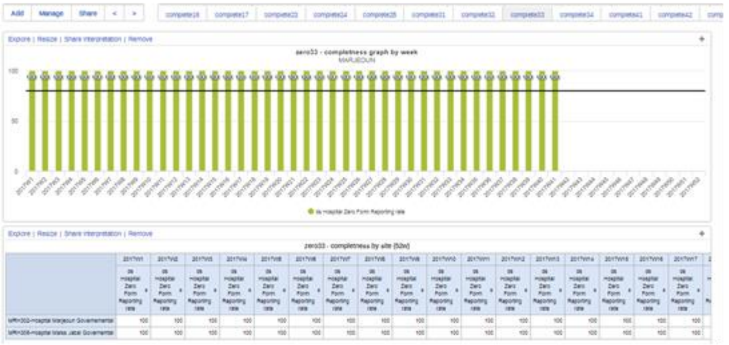

Generic dashboard for health facility

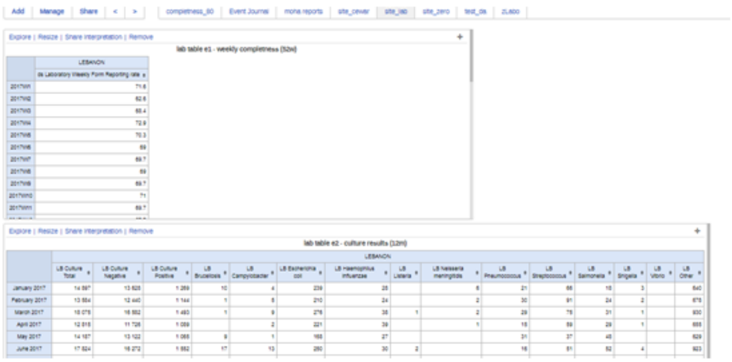

Supplement: Supplementary file 1 — Additional file 1. [file 12913_2022_7773_MOESM1_ESM.pdf]
